# Using in silico predicted ancestral genomes to improve the efficiency of paleogenome reconstruction

**DOI:** 10.1002/ece3.6925

**Published:** 2020-10-28

**Authors:** Filipe Garrett Vieira, José Alfredo Samaniego Castruita, M. Thomas P. Gilbert

**Affiliations:** ^1^ Section for Evolutionary Genomics The GLOBE Institute Faculty of Health and Medical Sciences University of Copenhagen Copenhagen Denmark; ^2^ University Museum Norwegian University of Science and Technology Trondheim Norway

**Keywords:** ancestral genome reconstruction, paleogenomics

## Abstract

Paleogenomics is the nascent discipline concerned with sequencing and analysis of genome‐scale information from historic, ancient, and even extinct samples. While once inconceivable due to the challenges of DNA damage, contamination, and the technical limitations of PCR‐based Sanger sequencing, following the dawn of the second‐generation sequencing revolution, it has rapidly become a reality. However, a significant challenge facing ancient DNA studies on extinct species is the lack of closely related reference genomes against which to map the sequencing reads from ancient samples. Although bioinformatic efforts to improve the assemblies have focused mainly in mapping algorithms, in this article we explore the potential of an alternative approach, namely using reconstructed ancestral genome as reference for mapping DNA sequences of ancient samples. Specifically, we present a preliminary proof of concept for a general framework and demonstrate how under certain evolutionary divergence thresholds, considerable mapping improvements can be easily obtained.

## Graphical abstract

A significant challenge facing ancient DNA studies on extinct species is the lack of closely related reference genomes against which to map the sequencing reads from the ancient samples. Although bioinformatic efforts to improve the assemblies have focused mainly in mapping algorithms, in this article we explore the potential of an alternative approach, namely using reconstructed ancestral genome as reference for mapping DNA sequences of ancient samples.

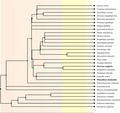

## 1 INTRODUCTION

The number and quality of sequenced genomes is rapidly increasing as DNA sequencing technologies become increasingly faster and cheaper per base sequence (Mardis, 2011). This has been revolutionary across the fields of biology, enabling researchers to not only introduce genomic approaches to model organisms, but across the tree of life (Ellegren, 2014). In parallel, the sequencing of genomes from ancient DNA (aDNA) within historic, ancient, or otherwise degraded samples has also started to become of growing interest, and these so‐called paleogenomic studies have helped us better understand the history of ancient species and populations (Brunson & Reich, 2019; MacHugh et al., 2017; Nielsen et al., 2017; Przelomska et al., 2020). Although DNA from such samples has played an important role in understanding species relationships, it is not without its limitations, since the molecules recovered are usually far from optimal for genome sequencing due to the challenges of *postmortem* damage (Hofreiter, Jaenicke, Serre, von Haeseler, & Pääbo, 2001; Hofreiter, Serre, et al., 2001; Lindahl, 1993). For instance, ancient samples may often have a low endogenous DNA content due to infiltration of DNA “contaminants” derived principally from microbial sources or have undergone *postmortem* degradation (Poinar et al., 2006). Although the exact contributions of individual processes to the damage will vary with the environment surrounding specimens (Hofreiter, Jaenicke, et al., 2001; Hofreiter, Serre, et al., 2001;), key processes include cross‐linking of DNA to other molecules in the surrounding matrix (Pääbo, 1989), fragmentation through the introduction of abasic sites or nicks in the phosphate backbone, and generation of miscoding lesions due principally to hydrolytic deamination (Lindahl, 1993). These DNA damage processes have considerable relevance for the potential of generating genomic sequences from ancient materials. At the most basic level, due to the degradation of DNA as a function of temperature and time (Lindahl, 1993; Smith et al., 2001), there is an ultimate time window beyond which no usable DNA can be recovered. While initially argued by many to lie in the range of tens to few‐100 thousand years ago (KYA) for temperate preserved material (Wayne et al., 1999), recent groundbreaking studies have pushed this window back to over 700 KYA in permafrozen bones (Orlando et al., 2013). Of further relevance, is the effect that this damage (and contaminating exogenous DNA) plays on the quality of genomes that can be sequenced from ancient samples. Endogenous DNA sequences recovered are predominantly <100 base pairs (bp) in length (Poinar et al., 2006); thus, the de novo assembly of extinct eukaryotic genomes—techniques that largely require long DNA fragments in order to better resolve sequence variation and regions of low complexity—is currently not possible (Kircher, 2012), even though some assemblies have been attempted on ancient microbial genomes and the Tasmanian tiger (the latter without much success (Feigin et al., 2018)). Thus, ancient genome reconstructions (whether of extinct or extant species) have been almost exclusively based around mapping sequence data to references. While powerful when a reference is closely related (e.g., ancient versus modern horse; Orlando et al., 2013), sometimes no closely related species exist, and even when they do, as evolutionary distance increases between an ancient sample and modern reference, so does the challenge of mapping the data and thus the percentage of endogenous DNA that can be mapped (Shapiro & Hofreiter, 2014). This effect can easily be exemplified using sequence data from modern genome projects, specifically through mapping raw sequence reads of one taxa against the genome of a related species. This was clearly shown in a seminal paper (Prüfer et al., 2010), where the authors demonstrated how, when mapping Neanderthal sequence reads to various mammal species, success rate dropped over tenfold as the reference moved from human (<1 million years [Mya.] divergence) to mouse (ca. 87 Mya. divergence). This pattern is likely consistent across the tree of life—for example, a simple in silico experiment reveals that only ca 5% of the raw Illumina 100bp sequence reads derived from the white‐throated tinamou genome published by the B10k project (https://b10k.genomics.cn/; Zhang et al., 2014) can be mapped to the genome of the ca 80 Mya. divergent ostrich using standard mapping software such as BWA (Li & Durbin, 2009), with mapping reads predictably located within the most conserved exons (Jose Samaniego Castruita, unpublished observation). The implications of such studies are threefold. Firstly, with an estimated divergence of 50–80 Mya. between these species and one of their extinct, high profile relative clades, the moas of New Zealand (Cooper et al., 2001), reconstructing a near complete genome in this way is going to be extremely challenging, if not impossible. Secondly, mapping‐based estimates of the endogenous DNA content of sequencing datasets generated through shotgun sequencing of extinct species are likely underestimates (a 1% mapping success rate of a moa sample, at estimated 5% overall mapping success, suggests an overall endogenous DNA content of 20%). Thirdly, as mappability directly correlates with the degree of conservation between the two genomes, any evolutionary relevant changes outside of the most conserved regions will not be recovered (Prüfer et al., 2010; Richmond et al., 2016).

Clearly, given the interest in the reconstruction of complete genomes of now extinct taxa, this is not an optimal situation for us to be in. If we are to accept the premise that we will be unable to improve the quality of the DNA recovered from many such samples, the question is raised as to whether there is any other possibility for improving the quality of genome recovered. In this regard, one potential might be through exploring computational approaches to deal with the particular challenge of the evolutionary divergence.

Using bioinformatic tools to improve the quality of data generation in paleogenomics is not a new approach, although to date most research has addressed this challenge by accounting for the miscoding lesions derived by postmortem damage that plague the ancient DNA molecules. Derived principally from the deamination of cytosines to uracil and its chemical analogues (Gilbert et al., 2003, 2007; Hansen et al., 2001; Hofreiter, Jaenicke, et al., 2001; Hofreiter, Serre, et al., 2001; Lindahl, 1993; Pääbo, 1989), represented as C‐>T and G‐>A mutations in the resulting sequence, and in particular concentrated at the 5′ and 3′ extremes of the templates sequenced (Briggs et al., 2007; Brotherton et al., 2007), these mismatches significantly reduce mapping efficacy (Schubert et al., 2012). Thus, several informatic‐based approaches exist, including rescaling base quality at sequence extremes to account for the damage probability (Jónsson et al., 2013), informed modification of alignment parameters (e.g., disable seed, or allowing for more mismatches; Orlando et al., 2015; Schubert et al., 2012), or adoption of aligners customized for aDNA, that do not assume uniform mismatches along the sequence read and enable some degree of sequence divergence between the reference and the target species. A number of probabilistic aligners based on position‐specific scoring matrices (PSSM) have been developed in this regard, such as MIA (Briggs et al., 2009), ANFO (Briggs et al., 2007), BWA‐PSSM (Kerpedjiev et al., 2014), and generally show good performance for short and/or low quality data. These can therefore improve the fraction of alignable hits, although some show running times only compatible with alignments against relatively small reference genomes (e.g., mitochondrial genomes). Furthermore, some of these approaches (e.g., allowing more mismatches, or PSSM mis‐specifications) can have unintended detrimental effects, leading to increased mapping errors. For this reason, accurate mapping against distantly related genomes is very challenging and, as a result, new mapping methods are required to improve the amount of genome recovered.

### 1.1 Use of ancestrally reconstructed genomes as reference

The growing availability of reference genomes from extant organisms indicates we are entering an era in which new mapping approaches should be considered for paleogenomics. While the above discussed approaches that aim to minimize the effects of miscoding lesions are useful, ultimately the evolutionary distance between extinct‐extant species remains the principal challenge (Richmond et al., 2016). In this regard, one theoretically attractive approach is reducing in silico this evolutionary distance, through ancestral genome reconstruction, a technique whose use has been explored at both the sequence and genomic architecture level. Embedded in particular in maximum parsimony (Fitch, 1971), maximum likelihood and Bayesian frameworks (Huelsenbeck & Bollback, 2001; Pagel, 1999; Pupko et al., 2000; Yang et al., 1995), ancestral sequence reconstruction (ASR) attempts to infer the state (amino acid or DNA) of the ancestral sequence through the use of modern sequences within a phylogenetic context (Randall et al., 2016). These have, for example, been used to reconstruct genomic traits such as the visual pigments of dinosaurs (Chang et al., 2002), the ancestral steroid hormone receptor (Thornton et al., 2003), alcohol dehydrogenases from yeast (Thomson et al., 2005), primate mitochondrial DNA (Krishnan et al., 2004), and over 500MB of the ancestral archosaur genome (Green et al., 2014). At the genomic architecture level, recent developments provide algorithms that handle repeats as well as diverse types of genome rearrangements and chromosome structures (Chauve & Tannier, 2008; Jones et al., 2012; Ma et al., 2006), and such methods have been used to reconstruct gene content (Cohen et al., 2010) and gene order, with the latter being used for reconstructing ancestral genomes organization of taxa ranging from bacteria (Fremez et al., 2007) to animals (Alekseyev & Pevzner, 2009; Green et al., 2014). In this study, we explore the potential use of ASR to reconstruct ancestral genomes and test their potential use as reference genomes, so as to improve the mapping of ancient samples.

### 1.2 Exploratory analysis

**FIGURE 1:**
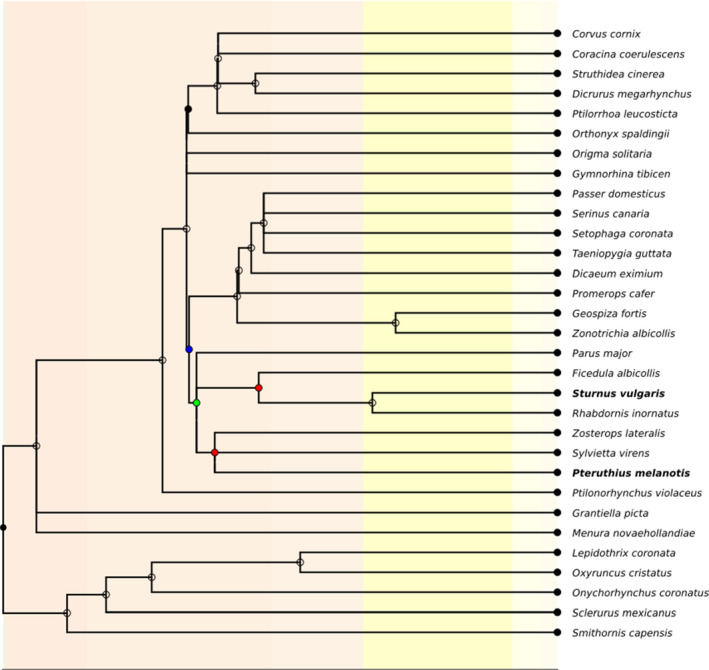
*Passeriformes* phylogeny used for sequence reconstruction. Test species are depicted in bold, and reconstructed ancestral nodes 1, 2, and 3 as colored circles (red, green, and blue, respectively)

Taking advantage of data generated recently by the Bird 10k Genome Consortium, kindly provided by Guojie Zhang and Shaohong Feng prior to its formal release (Feng, et al., in press), we selected 31 Passeriformes species whose genomes have been de novo sequenced and assembled (Figure 1 and Table 1), and aligned them using the zebra finch (*Taeniopygia guttata*) high quality genome as anchor (Warren et al., 2010). Since ASR is highly dependent on divergence times, we selected two test species with different divergence times to their respective sister species: *Sturnus vulgaris* (diverged from its closest sister species, *Rhabdornis inornatus*, ~20 Myra) and *Pteruthius melanotis* (diverged from its closest sister species, *Sylvietta virens*, ~40 Myra) (Kumar et al., 2017). For each one of these cases, we removed the test species from the alignment and reconstructed the ancestral sequences.

To evaluate the effects of using an ASR as reference, we assessed the mappability of simulated reads to several genomes: (i) test species reference genome, (ii) closest sister species reference genome, (iii) alignable/conserved fraction of the closest sister species reference genome, (iv) alignable/conserved fraction of reconstructed ancestral sequences (from 3 ancestral nodes), and (v) hybrid reference genomes between (ii) and (iv).

## 2 METHODS

### 2.1 Whole‐genome alignment

Whole‐genome alignments were created with the LASTZ + MULTIZ (Blanchette, 2004) pipeline across the 31 bird species chosen (Table 1), using the zebra finch genome as the anchor genome. We generated pairwise whole‐genome alignments between each bird species and the zebra finch using LASTZ (“‐‐step = 19 ‐‐hspthresh = 2,200 ‐‐gappedthresh = 10,000 ‐‐ydrop = 3,400 ‐‐inner = 2000 ‐‐seed = 12of19 ‐‐format = axt ‐‐scores = HoxD70”) and Chain/Net package (default parameters, except for axtChain where we used "‐minScore = 3,000 ‐linearGap = medium"). To prevent multiple hits from the whole‐genome alignment being used, we filtered out multiple reciprocal best hits keeping only the best. Since some alignment segments were considerably small, we filtered out segments using mafFilter (“‐factor ‐minCol = 100”). Lastly, to combine all LASTZ alignments, we used MULTIZ to merge pairwise alignments iteratively, according to the Passeriformes tree topology from TimeTree (www.timetree.org).

**TABLE 1:** List of bird species used in this study

Family	Genus	Species	Common Name	B10K ID	Species ID
Estrildidae	Taeniopygia	*Taeniopygia guttata*	Zebra Finch	NCBI‐013	TAEGU
Passeridae	Passer	*Passer domesticus*	House Sparrow	NCBI‐021	PASDO
Paridae	Parus	*Parus major*	Great Tit	NCBI‐022	PARMA
Muscicapidae	Ficedula	*Ficedula albicollis*	Collared Flycatcher	NCBI‐012	FICAL
Fringillidae	Serinus	*Serinus canaria*	Atlantic Canary	NCBI‐018	SERCA
Parulidae	Setophaga	*Setophaga coronata*	Yellow‐rumped Warbler	NCBI‐010	SETCO
Thraupidae	Geospiza	*Geospiza fortis*	Medium Ground Finch	APP‐023	GEOFO
Passerellidae	Zonotrichia	*Zonotrichia albicollis*	White‐throated Sparrow	NCBI‐007	ZONAL
Zosteropidae	Zosterops	*Zosterops lateralis*	Silver‐eye	NCBI‐003	ZOSLA
Sturnidae	Sturnus	*Sturnus vulgaris*	Common Starling	NCBI‐002	STUVU
Sylviidae	Sylvietta	*Sylvietta virens*	Green Crombec	B10K‐DU‐009‐59	SYLVI
Nectariniidae	Promerops	*Promerops cafer*	Cape Sugarbird	B10K‐UC‐030‐53	PROCA
Dicaeidae	Dicaeum	*Dicaeum eximium*	Red‐banded Flowerpecker	B10K‐DU‐001‐34	DICEX
Rhabdornithidae	Rhabdornis	*Rhabdornis inornatus*	Stripe‐breasted Rhabdornis	B10K‐DU‐001‐29	RHAIN
Timaliidae	Pteruthius	*Pteruthius melanotis*	Black‐eared Shrike‐babbler	B10K‐IZ‐033‐77	PTEME
Corvidae	Corvus	*Corvus cornix*	Hooded Crow	NCBI‐020	CORCO
Pipridae	Lepidothrix	*Lepidothrix coronata*	Blue‐crowned Manakin	NCBI‐008	LEPCO
Tyrannidae	Onychorhynchus	*Onychorhynchus coronatus*	Royal Flycatcher	B10K‐DU‐028‐75	ONYCO
Menuridae	Menura	*Menura novaehollandiae*	Superb Lyrebird	B10K‐CU‐030‐46	MENNO
Eurylaimidae	Smithornis	*Smithornis capensis*	African Broadbill	B10K‐CU‐031‐20	SMICA
Campephagidae	Edolisoma/ Coracina	*Coracina coerulescens*	Blackish Cicadabird	B10K‐DU‐001‐25	EDOCO
Orthonychidae	Orthonyx	*Orthonyx spaldingii*	Chowchilla	B10K‐DU‐029‐32	ORTSP
Acanthizidae	Origma	*Origma solitaria*	Rock‐warbler	B10K‐DU‐029‐52	ORISO
Ptilonorhynchidae	Ptilonorhynchus	*Ptilonorhynchus violaceus*	Satin Bowerbird	B10K‐DU‐‐012‐10	PTIVI
Cinclosomatidae	Ptilorrhoa	*Ptilorrhoa leucosticta*	Spotted Jewel‐babbler	B10K‐CU‐031‐17	PTILE
Cracticidae	Gymnorhina	*Gymnorhina tibicen*	Australian Magpie	B10K‐DU‐002‐05	GYMTI
Cotingidae	Oxyruncus	*Oxyruncus cristatus*	Sharpbill	B10K‐DU‐002‐07	OXYCR
Meliphagidae	Grantiella	*Grantiella picta*	Painted Honeyeater	B10K‐DU‐029‐50	GRAPI
Corcoracidae	Struthidea	*Struthidea cinerea*	Apostlebird	B10K‐DU‐029‐33	STRCI
Furnariidae	Sclerurus	*Sclerurus mexicanus*	Tawny‐throated Leaftosser	B10K‐DU‐001‐03	SCLME
Dicruridae	Dicrurus	*Dicrurus megarhynchus*	Ribbon‐tailed Drongo	B10K‐DU‐001‐48	DICME

Note

Columns stand for the species taxonomy (first three columns), common name, the “Bird 10,000 Genomes Project” ID (B10K ID, www.b10k.genomics.cn) and our own internal ID (Species ID).

### 2.2 Ancestral sequence reconstruction

Due to computational constraints, we excluded some of the outgroup species in our alignment since they would be unlikely to affect the reconstruction. That meant that, for our ancestral sequence reconstruction, only the first 15 species in Table 1 were used. To reconstruct the ancestral sequences from our species, we used RAxML v8.2.11 (Stamatakis, 2014). Briefly, and since we used the TimeTree (www.timetree.org) tree topology for the MULTIZ multiple‐alignment phase, we decided to use the same topology as a fixed parameter, and only estimate its branch lengths (“‐f e ‐m GTRGAMMA ‐t Passeriformes_species.B10k.nwk”); for computational reasons, and assuming it to be representative of the entire genome, only the largest chromosome (chr1) was used on this step. Then, using the tree topology from TimeTree with our estimated branch lengths, we again used RAxML to estimate the ancestor posterior probability distribution at each internal node, and choose the allele with the highest probability (“‐m GTRGAMMAX ‐‐HKY85”). All missing positions, as well as those with more than 90% missing data in the multiple sequence alignment, were removed from the ancestral reconstructed sequence.

### 2.3 Hybrid reference sequence

Since only a fraction of the genome is alignable between all 31 species, we tried to further improve mappability by creating a hybrid genome between each of the ancestrally reconstructed genomes and the, respectively, closest sister species of each test species. For that, we replaced all alignable parts of the sister species reference genome, with each of the ancestral reconstructed sequences.

### 2.4 Evaluation of reconstruction efficiency

To evaluate the mapping improvement between the several methods, we used wgsim v0.3.2 (https://github.com/lh3/wgsim) to simulate 10 million 100bp paired‐end reads with an error rate of 0%. These reads were then mapped using BWA‐MEM v0.7.15‐r1140 (Li & Durbin, 2009) with default parameters plus option “‐M,” against the sequences from each method. The reconstruction efficiency was then assessed from the percentage of total mapped reads, percentage of covered genome (where depth ≥ 1X), and average sequencing depth, excluding reads with a mapping quality lower than 20, not primary alignments (0 × 100), failing platform/vendor quality checks (0 × 200), or supplementary alignments (0 × 800); for that we used the program Mosdepth v0.2.6 (Pedersen & Quinlan, 2018).

## 3 RESULTS AND DISCUSSION

Our results as summarized in Table 2 show that, as expected, the sister species is not always a good reference genome. For short evolutionary distances (20 Mya.), it serves as a good proxy with only a slightly lower mapping efficiency than the original genome, as inferred from the percentage of mapped reads (95% vs. 99%), percentage of covered genome (78% vs. 83%), and average depth (1.74X vs. 1.96X). However, the performance drops notably with increasing distances (40 Mya.), with lower percentage of mapped reads (71% vs. 99%), covered genome (58% vs. 85%), and average depth (1.14X vs. 1.94X).

**TABLE 2:** Mapping efficiency across all tested scenarios

	*Pteruthius melanotis* (40 Mya)					*Sturnus vulgaris* (20 Mya)				
	Total length	Missing data	Mapped reads (%)	Bases ≥ 1X (%)	Avg. depth (X)	Total length	Missing data	Mapped reads (%)	Bases ≥ 1X (%)	Avg. depth (X)
Test Species Reference Genome	1,046,455,532	17,596,582	99.63	85.00	1.94	1,036,755,994	23,939,528	99.30	85.19	1.96
Sister Sp. Reference Genome	1,028,067,230	20,522,208	70.95	58.22	1.14	1,042,126,594	6,498,212	94.82	78.40	1.74
Sister Sp. (aligned fraction)	732,622,680	19,773	55.71	62.55	1.24	736,773,827	8,873	71.18	81.32	1.81
Ancestral Node 1 (aligned fraction)	770,705,063	0	57.07	61.68	1.22	773,101,043	0	71.62	78.34	1.71
Ancestral Node 2 (aligned fraction)	792,655,734	0	67.52	71.44	1.51	792,123,549	0	72.45	77.33	1.70
Ancestral Node 3 (aligned fraction)	794,901,605	0	68.74	72.58	1.55	794,210,006	0	72.46	77.14	1.70
Ancestral Node 1 (hybrid)	1,034,141,039	20,389,813	70.93	58.55	1.15	1,048,788,001	6,429,009	93.85	77.14	1.70
Ancestral Node 2 (hybrid)	1,040,695,577	20,389,813	80.42	66.30	1.38	1,055,625,192	6,429,009	94.48	77.08	1.96
Ancestral Node 3 (hybrid)	1,041,423,848	20,389,813	81.50	67.18	1.41	1,056,312,809	6,429,009	94.47	77.00	1.70

Note

The table shows the total length of the reference genome, its amount of missing bases, percentage of mapped reads, percentage of bases covered by at least one read (excluding reference missing regions), and average depth across both test species and all tested reconstruction scenarios.

If the genetic distance between our paleospecies and the closest sister species is big, then we hypothesized that the use of an ancestrally reconstructed genome as reference should improve mapping efficiency. At first sight, this does not seem to be true, since these reconstructed genomes resulted in a lower percentage of mapped reads than obtained when using the sister species' or the reference genome (57%, 68%, and 69% vs. 71% or 99.6%). However, we believe this is due to the incompleteness of the reconstructed genomes, since only a fraction of the original 31 genomes can actually be aligned and, consequently, is possible to infer the ancestral state from. If we just take these conserved regions into account, we can clearly see that ancestrally reconstructed genomes show higher mapping efficiencies than the correspondent sister genome, especially for longer evolutionary distances, with higher percentage of mapped reads (57%, 68%, and 69% vs. 56%), covered genome (62%, 71%, and 73% vs. 63%), and average depth (1.22X, 1.51X, and 1.55X vs. 1.24X); at short evolutionary rates the increase is marginal, reflecting the high similarity of the sequences. Interestingly, the mapping efficiency increases considerably with evolutionary distance probably due to the fact that, at deep ancestral nodes, more information is used and so a more complete reconstruction is possible (reflected in longer sequences).

In order to improve the apparent lower mapping efficiency of the ancestrally reconstructed genomes, we devised one last approach where we created a hybrid sequence between them and the sister species genome. With this approach, we hoped to mitigate the effect of incomplete genome reconstruction, by using the sister species reference genomes where ancestral information was not available. This new approach greatly improved mapping efficiency, with the best case scenario largely surpassing the sister genome on all metrics: percentage of mapped reads (82% vs. 71%), percentage of covered genome (67% vs. 58%), and average depth (1.41X vs. 1.14X); at shorter distances, as seen above, the improvement was marginal or nonexistent.

## 4 CONCLUSION

We are entering an era where new approaches should be considered for paleogenomics in light of the growing interest in sequencing the genomes of now extinct species. While considerable effort has been dedicated to account for the effects of miscoding lesions (e.g., Jónsson et al., 2013) on the accuracy of the reconstructed ancient genomes, we believe that ultimately the evolutionary distance that separates extinct from extant relative species will remain a greater challenge. Here, we have explored the possibility of reducing this distance in silico by ancestral genome reconstruction.

Overall, our results show that ancestral genome reconstruction can be helpful at reducing the distance between extinct and extant species in silico, in particular under the “hybrid” genome approach. However, at the same time, our results also highlight some of the drawbacks associated with such an approach. For example, it requires a relatively high density of extant species around the paleogenome of interest, if no close sister species is available. This raises the obvious question of, how close is close enough? Unfortunately, given that the rate of genome evolution is not constant throughout the tree of life, we do not believe it is possible to give a clear answer to this question and feel that it will need to be explored case‐by‐case. However, we note that in the context of birds, divergences of the order of ca 20 My (or more) are clearly relevant for iconic taxa such as the moas (mitogenome based estimate of ca. 52.7 Mya from the tinamous; Grealy et al., 2017), elephant birds (mitogenome based estimate of ca. 53.5 Mya from the kiwis; Grealy et al., 2017), dodo (mitogenome based estimate of ca. 18 Mya divergence from the Nicobar pigeon; Soares et al., 2016), and great auk (mitochondrial fragment estimate of ca. 25 Mya from the razorbill; Pereira & Baker, 2008), all of which are both actively being discussed as candidates for genome reconstruction and even de‐extinction.

This work was intended as a preliminary proof of concept and, as so, we can foresee several possible improvement venues. Firstly, we acknowledge that our analysis was limited to birds, whose genomes are not only relatively small compared to those of many other vertebrates, but also well known for their degree of synteny conservation (Zhang et al., 2014). Given that our aim was to improve mappability (as opposed to de novo assembly), we suspect that is unlikely to affect the results; however, future analyses could be done using other taxa to explore this. Secondly, we note that, unlike our in silico data, true ancient DNA extracts contain molecules that are fragmented and thus span a range of template sizes, most of which are very short, and can contain DNA damage driven miscoding lesions (Poinar et al., 2006). While both would affect the efficiency of mapping the data to the reference genomes, including modifying the coverages obtained and possibly even ultimately incurring biases in population genomic analyses done using the genome (Gopalakrishnan et al., 2017; Orlando et al., 2013)—and certainly it might be interesting for future studies to explore this effect in detail, for example, simulating damaged DNA using software such as gargammel (Renaud et al., 2017)—we do not believe that this fundamentally changes our observation that ancestral genome reconstruction has a role to play when mapping deeply divergent taxa to a reference. Thirdly, and probably the most limiting step, improving the multiple sequence alignment between extant species can dramatically increase the fraction of aligned genomic regions and, as such, the genome fraction that can be reconstructed (Leimeister et al., 2019). Lastly, the ancestral inference step might also have room for improvements, by using, for example, more complex Maximum Likelihood (Yang et al., 1995) or Bayesian (Bouckaert et al., 2014; Lartillot et al., 2009) methods and models, especially those using marginal ancestral state reconstruction using the “classical” empirical Bayesian method that uses all sequences in the tree.

## CONFLICT OF INTERESTS

The authors declare no competing interests.

## AUTHOR CONTRIBUTION

**Filipe Garrett Vieira:** Data curation (lead); Formal analysis (lead); Investigation (lead); Methodology (lead); Project administration (lead); Resources (lead); Software (lead); Supervision (lead); Validation (lead); Visualization (lead); Writing‐original draft (lead); Writing‐review & editing (lead). **Jose Alfredo Samaniego Castruita:** Data curation (supporting); Formal analysis (supporting); Investigation (supporting); Methodology (supporting); Resources (supporting); Software (supporting); Validation (supporting); Visualization (supporting). **M. Thomas P. Gilbert:** Conceptualization (lead); Funding acquisition (lead); Project administration (lead); Supervision (supporting); Writing‐original draft (supporting); Writing‐review & editing (supporting).

## Data Availability

All data and software used in this study are already in the public domain.

## References

[ece36925-bib-0001] Alekseyev, M. A. , & Pevzner, P. A. (2009). Breakpoint graphs and ancestral genome reconstructions. Genome Research, 19(5), 943–957. 10.1101/gr.082784.108 19218533 PMC2675983

[ece36925-bib-0002] Blanchette, M. (2004). Aligning multiple genomic sequences with the threaded blockset aligner. Genome Research, 14(4), 708–715. 10.1101/gr.1933104 15060014 PMC383317

[ece36925-bib-0003] Bouckaert, R. , Heled, J. , Kühnert, D. , Vaughan, T. , Wu, C.‐H. , Xie, D. , Suchard, M. A. , Rambaut, A. , & Drummond, A. J. (2014). BEAST 2: A software platform for Bayesian evolutionary analysis. PLoS Computational Biology, 10(4), e1003537. 24722319 10.1371/journal.pcbi.1003537 PMC3985171

[ece36925-bib-0004] Briggs, A. W. , Good, J. M. , Green, R. E. , Krause, J. , Maricic, T. , Stenzel, U. , Lalueza‐Fox, C. , Rudan, P. , Brajkovic, D. , Kucan, Z. , Gusic, I. , Schmitz, R. , Doronichev, V. B. , Golovanova, L. V. , de la Rasilla, M. , Fortea, J. , Rosas, A. , & Paabo, S. (2009). Targeted retrieval and analysis of five Neandertal mtDNA genomes. Science, 325(5938), 318–321. 10.1126/science.1174462 19608918

[ece36925-bib-0005] Briggs, A. W. , Stenzel, U. , Johnson, P. L. F. , Green, R. E. , Kelso, J. , Prufer, K. , Meyer, M. , Krause, J. , Ronan, M. T. , Lachmann, M. , & Paabo, S. (2007). Patterns of damage in genomic DNA sequences from a Neandertal. Proceedings of the National Academy of Sciences of the United States of America, 104(37), 14616–14621. 10.1073/pnas.0704665104 17715061 PMC1976210

[ece36925-bib-0006] Brotherton, P. , Endicott, P. , Sanchez, J. J. , Beaumont, M. , Barnett, R. , Austin, J. , & Cooper, A. (2007). Novel high‐resolution characterization of ancient DNA reveals C > U‐type base modification events as the sole cause of post mortem miscoding lesions. Nucleic Acids Research, 35(17), 5717–5728. 17715147 10.1093/nar/gkm588 PMC2034480

[ece36925-bib-0007] Brunson, K. , & Reich, D. (2019). The promise of paleogenomics beyond our own species. Trends in Genetics: TIG, 35(5), 319–329. 10.1016/j.tig.2019.02.006 30954285

[ece36925-bib-0008] Chang, B. S. W. , Jönsson, K. , Kazmi, M. A. , Donoghue, M. J. , & Sakmar, T. P. (2002). Recreating a functional ancestral archosaur visual pigment. Molecular Biology and Evolution, 19(9), 1483–1489. 10.1093/oxfordjournals.molbev.a004211 12200476

[ece36925-bib-0009] Chauve, C. , & Tannier, E. (2008) A methodological framework for the reconstruction of contiguous regions of ancestral genomes and its application to mammalian genomes. PLoS Computational Biology, 4, e1000234. 19043541 10.1371/journal.pcbi.1000234 PMC2580819

[ece36925-bib-0010] Cohen, O. , Ashkenazy, H. , Belinky, F. , Huchon, D. , & Pupko, T. (2010). GLOOME: Gain loss mapping engine. Bioinformatics, 26(22), 2914–2915. 10.1093/bioinformatics/btq549 20876605

[ece36925-bib-0011] Cooper, A. , Lalueza‐Fox, C. , Anderson, S. , Rambaut, A. , Austin, J. , & Ward, R. (2001). Complete mitochondrial genome sequences of two extinct moas clarify ratite evolution. Nature, 409, 704–707. 10.1038/35055536 11217857

[ece36925-bib-0012] Ellegren, H. (2014). Genome sequencing and population genomics in non‐model organisms. Trends in Ecology & Evolution, 29(1), 51–63. 10.1016/j.tree.2013.09.008 24139972

[ece36925-bib-0013] Feigin, C. Y. , Newton, A. H. , Doronina, L. , Schmitz, J. , Hipsley, C. A. , Mitchell, K. J. , Gower, G. , Llamas, B. , Soubrier, J. , Heider, T. N. , Menzies, B. R. , Cooper, A. , O'Neill, R. J. , & Pask, A. J. (2018). Genome of the Tasmanian tiger provides insights into the evolution and demography of an extinct marsupial carnivore. Nature Ecology & Evolution, 2(1), 182–192. 10.1038/s41559-017-0417-y 29230027

[ece36925-bib-0014] Feng, S. , Stiller, J. , Deng, Y. , Armstrong, J. , Fang, Q. , Xie, D. , Chen, G. , Paten, B. , & Zhang, G. (in press). Densely sampling genomes across the diversity of birds increases power of comparative genomics analyses. Nature.

[ece36925-bib-0015] Fitch, W. M. (1971). Toward defining the course of evolution: Minimum change for a specific tree topology. Systematic Biology, 20(4), 404–416. 10.1093/sysbio/20.4.406

[ece36925-bib-0016] Fremez, R. , Faraut, T. , Fichant, G. , Gouzy, J. , & Quentin, Y. (2007). Phylogenetic exploration of bacterial genomic rearrangements. Bioinformatics, 23(9), 1172–1174. 10.1093/bioinformatics/btm070 17332021

[ece36925-bib-0017] Gilbert, M. T. P. , Binladen, J. , Miller, W. , Wiuf, C. , Willerslev, E. , Poinar, H. , Carlson, J. E. , Leebens‐Mack, J. H. , & Schuster, S. C. (2007). Recharacterization of ancient DNA miscoding lesions: Insights in the era of sequencing‐by‐synthesis. Nucleic Acids Research, 35(1), 1–10. 10.1093/nar/gkl483 16920744 PMC1802572

[ece36925-bib-0018] Gilbert, M. T. P. , Hansen, A. J. , Willerslev, E. , Rudbeck, L. , Barnes, I. , Lynnerup, N. , & Cooper, A. (2003). Characterization of genetic miscoding lesions caused by postmortem damage. American Journal of Human Genetics, 72(1), 48–61. 10.1086/345379 12489042 PMC420012

[ece36925-bib-0019] Gopalakrishnan, S. , Samaniego Castruita, J. A. , Sinding, M.‐H. , Kuderna, L. F. K. , Räikkönen, J. , Petersen, B. , Sicheritz‐Ponten, T. , Larson, G. , Orlando, L. , Marques‐Bonet, T. , Hansen, A. J. , Dalén, L. , & Gilbert, M. T. P. (2017). The wolf reference genome sequence (*Canis lupus lupus*) and its implications for *Canis* spp. population genomics. BMC Genomics, 18(1), 495 10.1186/s12864-017-3883-3 28662691 PMC5492679

[ece36925-bib-0020] Grealy, A. , Phillips, M. , Miller, G. , Gilbert, M. T. P. , Rouillard, J.‐M. , Lambert, D. , Bunce, M. , & Haile, J. (2017). Eggshell palaeogenomics: Palaeognath evolutionary history revealed through ancient nuclear and mitochondrial DNA from Madagascan elephant bird (*Aepyornis* sp.) eggshell. Molecular Phylogenetics and Evolution, 109, 151–163. 10.1016/j.ympev.2017.01.005 28089793

[ece36925-bib-0021] Green, R. E. , Braun, E. L. , Armstrong, J. , Earl, D. , Nguyen, N. , Hickey, G. , Vandewege, M. W. , St, J. A. , John, S.‐ C.‐G. , Castoe, T. A. , Kern, C. , Fujita, M. K. , Opazo, J. C. , Jurka, J. , Kojima, K. K. , Caballero, J. , Hubley, R. M. , Smit, A. F. , & Platt, R. N. , … Ray, D. A. (2014). Three crocodilian genomes reveal ancestral patterns of evolution among archosaurs. Science, 346(6215), 1254449. 25504731 10.1126/science.1254449 PMC4386873

[ece36925-bib-0022] Hansen, A. J. , Willerslev, E. , Wiuf, C. , Mourier, T. , & Arctander, P. (2001). Statistical evidence for miscoding lesions in ancient DNA templates. Molecular Biology and Evolution, 18(2), 262–265. 10.1093/oxfordjournals.molbev.a003800 11158385

[ece36925-bib-0023] Hofreiter, M. , Jaenicke, V. , Serre, D. , von Haeseler, A. , & Pääbo, S. (2001). DNA sequences from multiple amplifications reveal artifacts induced by cytosine deamination in ancient DNA. Nucleic Acids Research, 29(23), 4793–4799. 10.1093/nar/29.23.4793 11726688 PMC96698

[ece36925-bib-0024] Hofreiter, M. , Serre, D. , Poinar, H. N. , Kuch, M. , & Pääbo, S. (2001). Ancient DNA. Nature Reviews Genetics, 2(5), 353–359. 10.1038/35072071 11331901

[ece36925-bib-0025] Huelsenbeck, J. P. , & Bollback, J. P. (2001). Empirical and hierarchical Bayesian estimation of ancestral states. Systematic Biology, 50(3), 351–366. 10.1080/106351501300317978 12116580

[ece36925-bib-0026] Jones, B. R. , Rajaraman, A. , Tannier, E. , & Chauve, C. (2012). ANGES: Reconstructing ANcestral GEnomeS maps. Bioinformatics, 28(18), 2388–2390. 10.1093/bioinformatics/bts457 22820205

[ece36925-bib-0027] Jónsson, H. , Ginolhac, A. , Schubert, M. , Johnson, P. L. F. , & Orlando, L. (2013). mapDamage2.0: Fast approximate Bayesian estimates of ancient DNA damage parameters. Bioinformatics, 29(13), 1682–1684. 10.1093/bioinformatics/btt193 23613487 PMC3694634

[ece36925-bib-0028] Kerpedjiev, P. , Frellsen, J. , Lindgreen, S. , & Krogh, A. (2014). Adaptable probabilistic mapping of short reads using position specific scoring matrices. BMC Bioinformatics, 15, 100. 24717095 10.1186/1471-2105-15-100 PMC4021105

[ece36925-bib-0029] Kircher, M. (2012). Analysis of high‐throughput ancient DNA sequencing data. Methods in Molecular Biology, 840, 197–228. 22237537 10.1007/978-1-61779-516-9_23

[ece36925-bib-0030] Krishnan, N. M. , Seligmann, Hervé , Stewart, C.‐B. , de Koning, A. P. J. , & Pollock, D. D. (2004). Ancestral sequence reconstruction in primate mitochondrial DNA: Compositional bias and effect on functional inference. Molecular Biology and Evolution, 21(10), 1871–1883. 10.1093/molbev/msh198 15229290

[ece36925-bib-0031] Kumar, S. , Stecher, G. , Suleski, M. , & Hedges, S. B. (2017). TimeTree: A resource for timelines, timetrees, and divergence times. Molecular Biology and Evolution, 1812–1819. 10.1093/molbev/msx116 28387841

[ece36925-bib-0032] Lartillot, N. , Lepage, T. , & Blanquart, S. (2009). PhyloBayes 3: A Bayesian software package for phylogenetic reconstruction and molecular dating. Bioinformatics, 25(17), 2286–2288. 10.1093/bioinformatics/btp368 19535536

[ece36925-bib-0033] Leimeister, C.‐A. , Dencker, T. , & Morgenstern, B. (2019). Accurate multiple alignment of distantly related genome sequences using filtered spaced word matches as anchor points. Bioinformatics, 35(2), 211–218. 10.1093/bioinformatics/bty592 29992260 PMC6330006

[ece36925-bib-0034] Li, H. , & Durbin, R. (2009). Fast and accurate short read alignment with Burrows‐Wheeler transform. Bioinformatics, 25(14), 1754–1760. 10.1093/bioinformatics/btp324 19451168 PMC2705234

[ece36925-bib-0035] Lindahl, T. (1993). Instability and decay of the primary structure of DNA. Nature, 362(6422), 709–715. 10.1038/362709a0 8469282

[ece36925-bib-0036] Ma, J. , Zhang, L. , Suh, B. B. , Raney, B. J. , Burhans, R. C. , Kent, W. J. , Blanchette, M. , Haussler, D. , & Miller, W. (2006). Reconstructing contiguous regions of an ancestral genome. Genome Research, 16(12), 1557–1565. 10.1101/gr.5383506 16983148 PMC1665639

[ece36925-bib-0037] MacHugh, D. E. , Larson, G. , & Orlando, L. (2017). Taming the past: Ancient DNA and the study of animal domestication. Annual Review of Animal Biosciences, 5, 329–351. 10.1146/annurev-animal-022516-022747 27813680

[ece36925-bib-0038] Mardis, E. R. (2011). A decade's perspective on DNA sequencing technology. Nature, 470(7333), 198–203. 21307932 10.1038/nature09796

[ece36925-bib-0039] Nielsen, R. , Akey, J. M. , Jakobsson, M. , Pritchard, J. K. , Tishkoff, S. , & Willerslev, E. (2017). Tracing the peopling of the world through genomics. Nature, 541(7637), 302–310. 10.1038/nature21347 28102248 PMC5772775

[ece36925-bib-0040] Orlando, L. , Gilbert, M. T. P. , & Willerslev, E. (2015). Reconstructing ancient genomes and epigenomes. Nature Reviews Genetics, 16(7), 395–408. 10.1038/nrg3935 26055157

[ece36925-bib-0041] Orlando, L. , Ginolhac, A. , Zhang, G. , Froese, D. , Albrechtsen, A. , Stiller, M. , Schubert, M. , Cappellini, E. , Petersen, B. , Moltke, I. , Johnson, P. L. F. , Fumagalli, M. , Vilstrup, J. T. , Raghavan, M. , Korneliussen, T. , Malaspinas, A.‐S. , Vogt, J. , Szklarczyk, D. , Kelstrup, C. D. , … Willerslev, E. (2013). Recalibrating Equus evolution using the genome sequence of an early Middle Pleistocene horse. Nature, 499(7456), 74–78. 10.1038/nature12323 23803765

[ece36925-bib-0042] Pääbo, S. (1989). Ancient DNA: Extraction, characterization, molecular cloning, and enzymatic amplification. Proceedings of the National Academy of Sciences of the United States of America, 86(6), 1939–1943. 10.1073/pnas.86.6.1939 2928314 PMC286820

[ece36925-bib-0043] Pagel, M. (1999). The maximum likelihood approach to reconstructing ancestral character states of discrete characters on phylogenies. Systematic Biology, 48(3), 612–622. 10.1080/106351599260184

[ece36925-bib-0044] Pedersen, B. S. , & Quinlan, A. R. (2018). Mosdepth: Quick coverage calculation for genomes and exomes. Bioinformatics, 34(5), 867–868. 10.1093/bioinformatics/btx699 29096012 PMC6030888

[ece36925-bib-0045] Pereira, S. L. , & Baker, A. J. (2008). DNA evidence for a Paleocene origin of the Alcidae (Aves: Charadriiformes) in the Pacific and multiple dispersals across northern oceans. Molecular Phylogenetics and Evolution, 46(2), 430–445. 10.1016/j.ympev.2007.11.020 18178108

[ece36925-bib-0046] Poinar, H. N. , Schwarz, C. , Qi, J. I. , Shapiro, B. , MacPhee, R. D. E. , Buigues, B. , Tikhonov, A. , Huson, D. H. , Tomsho, L. P. , Auch, A. , Rampp, M. , Miller, W. , & Schuster, S. C. (2006). Metagenomics to paleogenomics: Large‐scale sequencing of mammoth DNA. Science, 311(5759), 392–394. 10.1126/science.1123360 16368896

[ece36925-bib-0047] Prüfer, K. , Stenzel, U. , Hofreiter, M. , Pääbo, S. , Kelso, J. , & Green, R. E. (2010). Computational challenges in the analysis of ancient DNA. Genome Biology, 11(5), R47. 20441577 10.1186/gb-2010-11-5-r47 PMC2898072

[ece36925-bib-0048] Przelomska, N. A. S. , Armstrong, C. G. , & Kistler, L. (2020). Ancient plant DNA as a window into the cultural heritage and biodiversity of our food system. Frontiers in Ecology and Evolution, 8, 74.

[ece36925-bib-0049] Pupko, T. , Pe, I. , Shamir, R. , & Graur, D. (2000). A fast algorithm for joint reconstruction of ancestral amino acid sequences. Molecular Biology and Evolution, 17(6), 890–896. 10.1093/oxfordjournals.molbev.a026369 10833195

[ece36925-bib-0050] Randall, R. N. , Radford, C. E. , Roof, K. A. , Natarajan, D. K. , & Gaucher, E. A. (2016). An experimental phylogeny to benchmark ancestral sequence reconstruction. Nature Communications, 7, 12847. 10.1038/ncomms12847 PMC5027606 27628687

[ece36925-bib-0051] Renaud, G. , Hanghøj, K. , Willerslev, E. , & Orlando, L. (2017). gargammel: A sequence simulator for ancient DNA. Bioinformatics, 33(4), 577–579. 27794556 10.1093/bioinformatics/btw670 PMC5408798

[ece36925-bib-0052] Richmond, D. J. , Sinding, M.‐H.‐S. , & Gilbert, M. T. P. (2016). The potential and pitfalls of de‐extinction. Zoologica Scripta, 45, 22–36. 10.1111/zsc.12212

[ece36925-bib-0053] Schubert, M. , Ginolhac, A. , Lindgreen, S. , Thompson, J. F. , Al‐Rasheid, K. A. S. , Willerslev, E. , Krogh, A. , & Orlando, L. (2012). Improving ancient DNA read mapping against modern reference genomes. BMC Genomics, 13(1), 178. 22574660 10.1186/1471-2164-13-178 PMC3468387

[ece36925-bib-0054] Shapiro, B. , & Hofreiter, M. (2014). A paleogenomic perspective on evolution and gene function: New insights from ancient DNA. Science, 343(6169), 1236573 10.1126/science.1236573 24458647

[ece36925-bib-0055] Smith, C. I. , Chamberlain, A. T. , Riley, M. S. , Cooper, A. , Stringer, C. B. , & Collins, M. J. (2001). Neanderthal DNA. Not just old but old and cold? Nature, 410(6830), 771–772. 10.1038/35071177 11298436

[ece36925-bib-0056] Soares, A. E. R. , Novak, B. J. , Haile, J. , Heupink, T. H. , Jon Fjeldså, M. , Gilbert, T. P. , Poinar, H. , Church, G. M. , & Shapiro, B. (2016). Complete mitochondrial genomes of living and extinct pigeons revise the timing of the columbiform radiation. BMC Evolutionary Biology, 16(1), 230. 27782796 10.1186/s12862-016-0800-3 PMC5080718

[ece36925-bib-0057] Stamatakis, A. (2014). RAxML version 8: A tool for phylogenetic analysis and post‐analysis of large phylogenies. Bioinformatics, 30(9), 1312–1313. 10.1093/bioinformatics/btu033 24451623 PMC3998144

[ece36925-bib-0058] Thomson, J. M. , Gaucher, E. A. , Burgan, M. F. , De Kee, D. W. , Li, T. , Aris, J. P. , & Benner, S. A. (2005). Resurrecting ancestral alcohol dehydrogenases from yeast. Nature Genetics, 37(6), 630–635. 10.1038/ng1553 15864308 PMC3618678

[ece36925-bib-0059] Thornton, J. , Need, E. , & Crews, D. (2003). Resurrecting the ancestral steroid receptor: Ancient origin of estrogen signaling. Science, 301(5640), 1714–1717. 10.1126/science.1086185 14500980

[ece36925-bib-0060] Warren, W. C. , Clayton, D. F. , Ellegren, H. , Arnold, A. P. , Hillier, L. W. , Künstner, A. , Searle, S. , White, S. , Vilella, A. J. , Fairley, S. , Heger, A. , Kong, L. , Ponting, C. P. , Jarvis, E. D. , Mello, C. V. , Minx, P. , Lovell, P. , Velho, T. A. F. , Ferris, M. , … Wilson, R. K. (2010). The genome of a songbird. Nature, 464(7289), 757. 20360741 10.1038/nature08819 PMC3187626

[ece36925-bib-0061] Wayne, R. K. , Leonard, J. A. , & Cooper, A. (1999). Full of sound and fury: History of ancient DNA. Annual Review of Ecology and Systematics, 30(1), 457–477. 10.1146/annurev.ecolsys.30.1.457

[ece36925-bib-0062] Yang, Z. , Kumar, S. , & Nei, M. (1995). A new method of inference of ancestral nucleotide and amino acid sequences. Genetics, 141(4), 1641–1650. 8601501 10.1093/genetics/141.4.1641 PMC1206894

[ece36925-bib-0063] Zhang, G. , Li, C. , Li, Q. , Li, B. , Larkin, D. M. , Lee, C. , Storz, J. F. , Antunes, A. , Greenwold, M. J. , Meredith, R. W. , Odeen, A. , Cui, J. , Zhou, Q. , Xu, L. , Pan, H. , Wang, Z. , Jin, L. , Zhang, P. , Hu, H. , … Froman, D. P. (2014). Comparative genomics reveals insights into avian genome evolution and adaptation. Science, 346(6215), 1311–1320. 10.1126/science.1251385 25504712 PMC4390078

